# Modelling the shape of the pig scapula

**DOI:** 10.1186/s12711-020-00555-5

**Published:** 2020-07-01

**Authors:** Øyvind Nordbø

**Affiliations:** 1 Norsvin SA, Storhamargata 44, 2317 Hamar, Norway; 2 Geno SA, Storhamargata 44, 2317 Hamar, Norway

## Abstract

**Background:**

The shape of pig scapula is complex and is important for sow robustness and health. To better understand the relationship between 3D shape of the scapula and functional traits, it is necessary to build a model that explains most of the morphological variation between animals. This requires point correspondence, i.e. a map that explains which points represent the same piece of tissue among individuals. The objective of this study was to further develop an automated computational pipeline for the segmentation of computed tomography (CT) scans to incorporate 3D modelling of the scapula, and to develop a genetic prediction model for 3D morphology.

**Results:**

The surface voxels of the scapula were identified on 2143 CT-scanned pigs, and point correspondence was established by predicting the coordinates of 1234 semi-landmarks on each animal, using the coherent point drift algorithm. A subsequent principal component analysis showed that the first 10 principal components covered more than 80% of the total variation in 3D shape of the scapula. Using principal component scores as phenotypes in a genetic model, estimates of heritability ranged from 0.4 to 0.8 (with standard errors from 0.07 to 0.08). To validate the entire computational pipeline, a statistical model was trained to predict scapula shape based on marker genotype data. The mean prediction reliability averaged over the whole scapula was equal to 0.18 (standard deviation = 0.05) with a higher reliability in convex than in concave regions.

**Conclusions:**

Estimates of heritability of the principal components were high and indicated that the computational pipeline that processes CT data to principal component phenotypes was associated with little error. Furthermore, we showed that it is possible to predict the 3D shape of scapula based on marker genotype data. Taken together, these results show that the proposed computational pipeline closes the gap between a point cloud representing the shape of an animal and its underlying genetic components.

## Background

The need for high-throughput phenotyping in animal breeding has accelerated the development of automated segmentation of computed tomography (CT) images [1–3] and the generation of new precision phenotypes based on the morphology of animals [4]. Detailed 3D models would be useful for the development of new indicators for economically important traits that are not directly measurable on selection candidates, such as longevity and robustness. Including such relevant indicator phenotypes in multi-trait genomic evaluations has the potential to increase the accuracy of estimated breeding values for traits under selection [4–6].

In human medicine, tremendous progress has already been achieved to create accurate descriptions of the shape of organs, bones, and surfaces through statistical shape modelling (SSM) [7]. More recently, the use of 3D cameras for web communication and entertainment, has accelerated the development of SSM of the face in humans [8]. In humans, SSM of the face has also been used in genetic studies that aimed at building methods for the identification of people from biological material [9, 10]. In livestock science, there is also a lot of new research that focuses on 3D imaging since it provides automatic and objective measures on live animals for breeding applications [11]. Much emphasis has been placed on replacement of phenotypes that, traditionally, have involved a large amount of manual work, for example weight [12–14], body condition score, and locomotion [15, 16]. Automatic high-throughput phenotyping from imaging technologies provides precise measures on already developed phenotypes, and simultaneously provides information about an individual from the 3D surface data. As such, 3D imaging has the potential to provide objective information about an individual’s condition and health. This could be used to gain a better understanding of the mechanisms that underlie animal health and robustness [4] and to develop relevant phenotypes for animal breeding [17].

The images recorded by, e.g., CT or 3D cameras are point clouds of surfaces and tissues without any annotation. To create relevant scalar phenotypes, which are the input data of the traditional mixed model equations used for animal breeding [18], these images must be annotated. The objects of interest may be rotated in relation to each other and are represented by different numbers of pixels. Hence, there is a need to create point correspondence [19], i.e. a map that explains which points represent the same piece of tissue among individuals.

In this paper, we report the construction of a computational pipeline for generating surface meshes with point correspondence based on automatic segmented CT images. Furthermore, we perform a principal component analysis (PCA) of the 3D shape and present a physical interpretation of the first principal components (PC). Finally, we build a statistical genomic prediction model for the 3D scapula surface and present the prediction accuracy of this model through a cross-validation study.

Because of its complex shape and its importance for sow robustness and health, we used the pig scapula as a model organ [4], but our computational pipeline can, in theory, be applied to any 3D object that represents the surface of an organ, bone, or body.

## Methods

### Animals

The animals in this study were purebred Landrace pigs that originated from the Norsvin breeding nucleus. All the phenotypes used were part of routine records in the breeding programme and conducted in accordance to the laws and legislations for raising pigs in Norway. In total, 2143 boars were CT-scanned at the boar testing station as part of the Topigs Norsvin breeding program [20]. The animals were born and raised to 25–30 kg in different nucleus herds located in Norway before being sent to the boar test station. The boar test included feed and weight records, scores for conformation traits, and CT scanning at the end of the test (at 120 kg) [21].

### Genotypes

Single nucleotide polymorphism (SNP) genotyping was performed at CIGENE (University of Life Sciences, Ås, Norway) or at GeneSeek (Lincoln, NE, USA), using either the Illumina GeneSeek custom 80 K SNP chip (Lincoln, NE, USA), the Illumina Porcine SNP60 Beadchip (Illumina Inc., San Diego, CA, USA), an Illumina GeneSeek custom 50 K SNP chip (Lincoln, NE, USA), or the Illumina Porcine SNP9 Beadchip (Illumina Inc., San Diego, CA, USA). Genome positions of the SNPs were based on the Sscrofa10.2 assembly of the reference genome [22]. The genotypes were filtered [23] and imputed using the AlphaImpute software [24, 25]. After imputation, genotypes on 34,726 SNPs were available for 2088 animals.

### Ethics approval

All animals were cared for according to the laws and regulations for keeping pigs in Norway (Regulation for the keeping of pigs in Norway 2003-02-18-175, 2003; Animal welfare Act 2009-06-19-97, 2009). In Norway, animal breeding is controlled by “Mattilsynet” (Norwegian Food Safety Authority), who has officially approved Norsvin for maintaining herd-books through article 1(d) of Directive 88/661/EEC (approval date 27.07.1994). The official herd ID for the boar test station is 0403050826. Data recording and sample collection were conducted strictly in line with the laws given by Norwegian animal research authorities on the protection of animals (“Lov om dyrevelferd”). The data were obtained as part of routine data recording in commercial breeding programs. Samples collected for DNA extraction were used only for the routine diagnostic purpose of the breeding program.

### Creation of phenotypes from CT-images

The full skeleton of live pigs was identified by applying a threshold value of 200 Hounsfield units to the CT scans [26]. Thereafter, the left scapula was identified and extracted [2], before modelling the 3D morphology of the scapula in Python, which involved the steps described below.

#### Creation of the scapula atlas

**Fig. 1:**
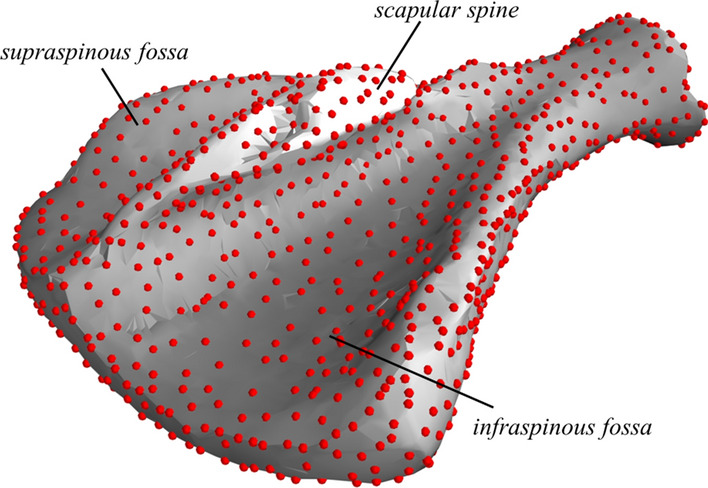
A dense surface mesh (shown in grey) of a CT-scanned purebred Norwegian Landrace scapula with semi-landmarks (shown as red dots)
- To reduce the computational load and to avoid problems with multiple point correspondence, when fitting semi landmarks (see (b) in “Point correspondence” below), a sparse subset of surface nodes was selected. This was done by going through all the nodes in the dense surface mesh and calculating the local volume of the sub-mesh (i.e. the mesh consisting of the convex hull of each node and the other nodes that shared faces with this node). For sub-meshes with volumes smaller than a certain threshold, the centre node was removed and a new local mesh was built. After removing nodes that were associated with the smallest local volumes, the remaining nodes constituted the scapula atlas. By doing this, the number of surface points was reduced from 7472 to 1234 semi-landmarks (red dots in Fig. 1).

For the sake of simplicity, the surface of the left scapula was modelled for a random pig with a scapula that looked normal by visual inspection of the CT image:

- The scapula was centred, rotated, and scaled to its second invariant (on scapula CT images, the width is more precisely measured than the length [4]).
- The surface voxels were extracted. As an alternative to the marching cubes algorithm [27], which also has the potential to create surface meshes from e.g. 3D camera images, a standard 3D Delaunay triangularization [28] was performed. This gives a convex volumetric mesh representation of the scapula. To also represent the concave regions, tetrahedrons, which had one or more edge longer than a specified threshold (5 mm), were removed. This is similar to the concept of alpha shapes and allows the object to have a partly concave surface. Inside the triangularization, all faces belong to two adjacent tetrahedrons, while surface faces belong to only one tetrahedron. To go from a volumetric mesh to a surface mesh representation of the scapula, faces that belonged to two tetrahedrons were removed, and only the nodes that belonged to the remaining (surface) faces were extracted and defined the surface of the object. The output from this step was a dense surface mesh (Fig. 1) that consisted of 7472 surface nodes, interpolated by 14,946 faces.

#### Point correspondence

**Fig. 2:**
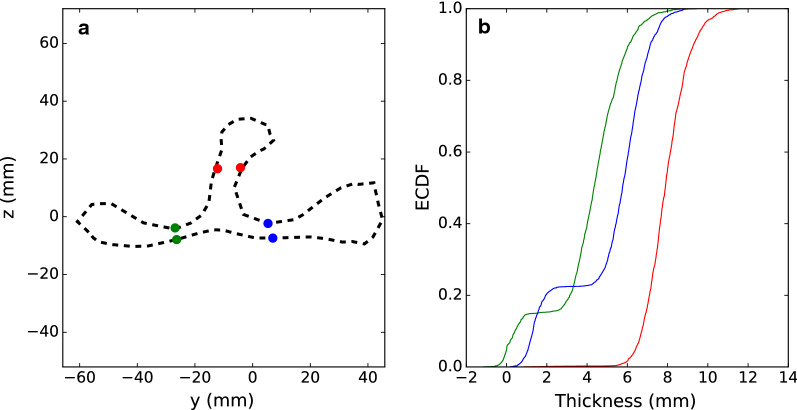
**a** A contour of the average pig scapula in the y–z plane for x-axis values between − 5 and +5 mm. The dots (red, blue and green) indicate points that were used to measure whether surface points were fitted on the wrong side of the bone. **b** Empirical Cummulative Distribution Function-plot of the distance between fitted point-pairs at opposite sides of the scapula surface. Green represents the distance between supraspinous fossa and subscapular fossa, blue the distance between infraspinous fossa and subscapular fossa, and red the width of scapular spine

- Each scapula was centred, rotated, and scaled in the same manner as was done for the atlas and a dense surface mesh was built for the individual, as in (b) in “Creating the scapula atlas” above, where the number of nodes varied between animals.
- To achieve the same number and same meaning of all points or phenotypes for all individuals, we used the coherent point drift (CPD) algorithm [29], which is implemented in Python [30]. This algorithm and improvements of this method have been widely used to achieve point correspondence for a variety of applications [31]. The CPD-algorithm finds the transformation that best aligns two point-clouds, X and Y, using Gaussian mixture models (GMM) through expectation–maximization (EM) optimization. The Y-points are considered as GMM centroids, whereas the X’s are data points to be fitted. At the optimum, the two point-clouds are aligned and the output is the probability correspondence matrix ${\mathbf{P}}$, which contains the probability that any point from Y corresponds to any of the points in X. In our case, we used the sparse atlas mesh as X [(c) in “Creating the scapula atlas”], and the dense surface mesh for each individual as Y [(a) in “Point correspondence”]. At the optimum, we used the most probable point correspondence for all points in X to find the corresponding point in Y. Hence, the raw phenotypes were a subset (1234 points) of the dense surface mesh Y. Within the CPD algorithm, we tested three types of deformation, i.e. rigid, affine, and non-rigid [29], on a subset of 115 individuals, using default parameters. To compare the effect of different deformation modes, we compared how well all points in the dense surface point cloud for each individual were represented by the aligned subset. Based on the results from this initial test, we proceeded on the entire dataset that consisted of 2143 animals, using the affine deformation option.
- To better align the fitted point clouds of 1234 x, y, and z-coordinates for each animal, the centring, scaling, and rotating procedure from (a) in “Creating the scapula atlas” was repeated. For breeding purposes, size of the scalpula is an interesting phenotype, and therefore, data was rescaled to its original size and measured in mm in an Eulerian coordinate space.
- Earlier attempts to achieve point correspondence for scapula, resulted in problems with point matching in regions where surfaces on different sides of the objects are close to each other. For example, at the scapula spine, the bone is quite thin and aligned points for some individuals were fitted to the wrong side of the bone. To test whether the surface points were taken from the correct side of the spine, we measured the difference in y-values between two node points on each side of the spine, with similar x and z coordinate values (red points in Fig. 2a). In addition, we made similar measurements on some of the thinnest parts of the scapula. On each side of the spine, we selected two pairs of points with similar x and y values and measured the distance in the z direction (green and blue points in Fig. 2a).

#### Generation of scalar phenotypes

- To remove outliers, we summed the absolute deviations from the population mean for all 3702 phenotypes (1234 x, y, and z coordinates). Animals with a summed absolute deviation that was more than 1.5 interquartile, ranges below the first quartile or above the third quartile were identified as outliers and removed from the dataset. After this step, 2088 animals remained in the dataset.
- Because the number of raw phenotypes was very large and the covariance between the variables was large, PCA [32] was used to capture the main variation between individuals. A set of raw phenotypes for 200 animals were masked before running the PCA, since these animals constituted the validation set. In the PCA transformation, the M (1888) × N (3702) matrix ${\mathbf{X}}$ was transformed into a set of linearly uncorrelated variables, so the phenotype vector for each animal, $m$, could be expressed as:
  1 $${\mathbf{X}}_{m} = \bar{X} + \mathop \sum \limits_{n = 1}^{N} \varvec{w}_{n} s_{nm} ,$$
  where $\bar{X}$ is the population mean of ${\mathbf{X}}$, $\varvec{w}$ are the principal component loadings or coefficients, and *s* are the principal component scores. The first few principal component loadings or coefficients ($\varvec{w}$) are the new uncorrelated variables that explained the main variation in the data and were used as the final aggregated phenotypes, while the principal component score $s_{nm}$ was used as the phenotypic value of individual $m$ for the new aggregated trait $n$.

### Creating a statistical model

Next, we estimated the genetic parameters for the PCA scores using best linear unbiased prediction (BLUP) [33] multi-trait animal models with the DMU software [34], similar to the approach used for CT traits in previous work [4]. This analysis was conducted using a pedigree that contained all animals with CT data and five generation of ancestors, for 7315 animals. To limit the genetic analyses to a feasible number of traits, we included only the first 10 principal components. The PCA score phenotypes $s_{nm}$ were modelled in a mult-trait genetic analysis as:

2 $$s_{ijklmn} = {\text{HY}}_{in} + {\text{BM}}_{jn} + {\text{PN}}_{kn} + \beta \times {\text{LW}}_{ln} + {\text{a}}_{mn} + {\text{e}}_{ijklmn} ,$$

with herd year (${\text{HY}}$) of the boar’s birth, birth month (${\text{BM}}$), and parity number of the dam (${\text{PN}}$) as fixed effects, the boar’s phenotype for live weight at scanning date (${\text{LW}}$) as a fixed covariate, and the additive genetic effect of the animal (${\text{a}}_{mn}$) and the residual (${\text{e}}_{ijklmn}$) as random effects. The numbers of levels for ${\text{HY}}$, ${\text{BM}}$, and ${\text{PN}}$ were 148, 12, and 4, respectively, while the covariate ${\text{LW }}$ had a mean of 122.8 kg and a standard deviation of 5.2 kg.

The genetic and phenotypic variance-covariances matrices ${\mathbf{G}}$ and ${\mathbf{P}}$ were further examined to identify genetically uncorrelated linear combinations of the 10 PC traits by calculating the eigenvectors ${\mathbf{v}}$ and eigenvalues $\lambda$ of the ${\mathbf{GP}}^{ - 1}$ matrix [35]. The eigenvector associated with the largest eigenvalue shows the direction of the shape space that is associated with the highest heritability estimate, whereas the eigenvector associated with the smallest eigenvalue shows the direction captured by the 10 principal components for which variation is the least associated with the underlying genetics.

### Cross-validation using a genomic prediction model

The SNP genotypes of 2088 animals (1888 in training and 200 in validation) were used to compute genomic relationships [36] with the program Gmatrix [37] as:

3 $${\mathbf{G}} = \frac{{{\mathbf{ZZ^{\prime}}}}}{{2\sum p_{j} (1 - p_{j} )}} ,$$

where ${\mathbf{Z}}$ is a matrix of standardised SNPs. Genotypes, with elements $Z_{ij} = I_{ij} - 2p_{j }$, where $I_{ij}$ is the number of the first allele that animal $i$ carries for SNP $j$, with an allele frequency of $p_{j }$. The genomic relationship matrix was fitted to the training data to predict genomic breeding values [38] using the statistical model described above (Eq. 2).

The phenotypes of the validation animals were masked, and the breeding values for all 2088 animals were estimated. Then, the genomic estimated breeding values (GEBV), ${\text{a}}_{mn}$ for each of the validation animals were added to the relevant solutions of the fixed effects in the model (${\text{HY}}_{in} + {\text{BM}}_{jn} + {\text{PN}}_{kn} + {{\beta }} \times {\text{LW}}_{ln}$) to constitute predicted PCA scores, $\hat{s}_{nm}$. Since the data were extracted from a commercial breeding programme, the experimental setup was somewhat unbalanced, which could have led to unreliable estimates of fixed effects. To cope with this, we removed animals from the validation set for which the number of observations for any of their estimates of fixed effects was less than 4. By multiplying predicted PCA scores, $\hat{s}_{nm}$, by the corresponding PCA-loadings, we predicted the 3702 raw phenotypes, using the 10 first PC, as:

4 $$\hat{\varvec{X}}_{\varvec{m}} = \bar{X} + \mathop \sum \limits_{n = 1}^{10} \varvec{w}_{n} \hat{s}_{nm} .$$

Goodness-of-fit of the shape was quantified by the mean Euclidean distance $d\left( {\varvec{x}_{m} ,\hat{\varvec{x}}_{m} } \right)$ between corresponding genomic predicted points, $\hat{\varvec{x}}_{m} ,$ and true CT-data, $\varvec{x}_{m} ,$ for each of the validation animals. To investigate predictive performance over the surface [10], we also measured the spatial prediction reliability, $R^{2}$, using the following formula:

5 $$R^{2} = 1 - \frac{{\frac{1}{{M_{v} }}\mathop \sum \nolimits_{m = 1}^{{M_{v} }} d\left( {\varvec{x}_{m} ,\hat{\varvec{x}}_{m} } \right)^{2} }}{{\frac{1}{{M_{v} }}\mathop \sum \nolimits_{m = 1}^{{M_{v} }} d\left( {\varvec{x}_{m} ,\bar{\varvec{x}}} \right)^{2} }} ,$$

where the numerator is the squared Euclidean distance between true and predicted coordinates, averaged over the $M_{v}$ validation animals, and the denominator is the mean squared Euclidean distance between observed and average coordinates values.

### Visualization of results

For 3D visualization of the PCA components, we used the Mayavi [39] package. First, isonormals that pointed outwards were calculated for all faces in the mean shape mesh, and the average of isonormals of adjacent faces was used to calculate the isonormal of the nodes. The PCA loadings for each point explain how much of the variability of the raw data is explained by each of the principal components. The loadings were organized into a vector field for the 1234 3D data points that represented the scapula. Taking the dot product between this vector field and the isonormals provided a scalar field that represented how variation in the current component affects morphological changes, perpendicular to the scapula surface. This allowed for the visualization of both inward and outward deformations in heatmaps.

## Results

### Selection of the best deformation type

To compare how the three types of deformation, rigid, affine, and non-rigid, worked within the coherent point drift (CPD) algorithm for the type of data analyzed here, an initial test was done on a subset of 115 animals. For this subset, the left scapula surface data consisted of between 7472 and 11,228 data points per animal. After deforming the atlas onto each individual’s data, a subset of 1234 node points was extracted. To consider how well these 1234 node points represented the surface points, we calculated the Euclidean distance between all surface points and the closest node points. The median and maximum of these distances were calculated for each individual and then averaged over all individuals for each deformation type. For affine deformations, the median and maximum distances were 2.39 and 9.13 mm. For non-rigid deformations, the corresponding numbers were 2.46 and 10.29 mm, while for rigid formations, the median and maximum distances were 2.43 and 9.60 mm. Based on these statistics, the affine deformation was considered to best represent the scapula surface and was then used for the entire dataset.

#### Point correspondence

The coherent point drift algorithm was run successfully on the data from all 2143 animals, of which 55 were identified as outliers and removed from further analysis. The pair-wise distance between pairs of node points (indicated by red, blue and green dots in Fig. 2a) on opposite sides of the scapula was measured for all animals and is shown as an empirical cumulative distribution function (ECDF) plot in Fig. 2b.

Figure 2 shows that the width at the narrowing on the scapula spine had a smooth uniform distribution with an average of 8 mm and a standard deviation of 1 mm, which indicates that surface points were fitted to the correct side for all animals. The distance for the two point-pairs on the shoulder blade had a bimodal distribution, indicating that, for about 15% and 25% of animals, surface modelling was not perfect for the *supraspinous fossa* (shown in green) and the *infraspinous fossa* (shown in blue), respectively.

### Principal components

**Fig. 3:**
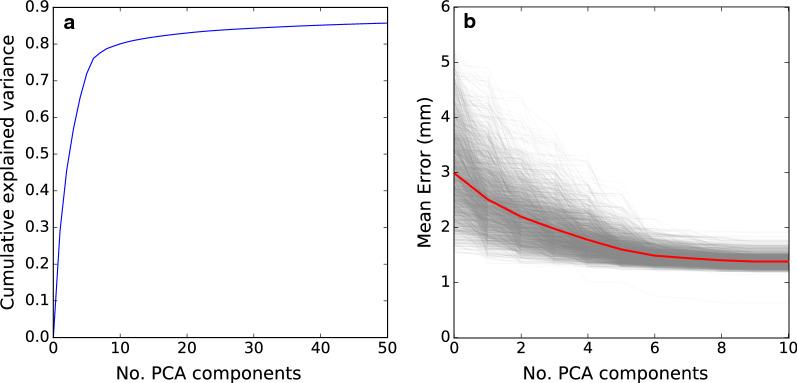
Cumulative variance explained by the 50 first principal components of the pig scapula (**a**), and mean error when comparing node positions based on the first 10 principal components with the true data (**b**).Grey lines represent the error for each animal and the red line represents the mean error across animals

Of the 2143 animals, 2088 passed outlier removal. Of these, data on 200 animals were masked before the PCA for validation purposes. The first three PC explained 29.1, 16.5, and 11.1% of the total variation, respectively, which means that more than 50% of the observed variation was captured by the first three PC, while more than 80% of the variation was captured by the first 10 PC (Table 1). After the first few components, the proportion of explained variance per PC shrank, and with 50 PC, 85.7% of the total variance was captured (Fig. 3a).

**Table 1:** Proportion of variance explained by the first 10 principal components (PC) (Exp. var.), and estimates of heritability (± SE) and of the regression coefficient, β, on live weight

PC	Exp. var.	$\varvec{h}^{2}$	SE	${\varvec{\upbeta}}$
PC1	0.29	0.52	0.07	− 0.23
PC2	0.17	0.56	0.08	− 1.16
PC3	0.11	0.68	0.07	0.16
PC4	0.09	0.67	0.07	0.64
PC5	0.07	0.50	0.07	− 0.25
PC6	0.04	0.63	0.07	0.19
PC7	0.02	0.78	0.07	− 0.31
PC8	0.01	0.79	0.07	− 0.07
PC9	0.01	0.68	0.07	− 0.03
PC10	0.01	0.40	0.07	0.10

*PC* principal components of the 3D scapula shape, $\beta$ = regression coefficient on live weight, correcting for the size of the animals in a genetic analysis

**Fig. 4:**
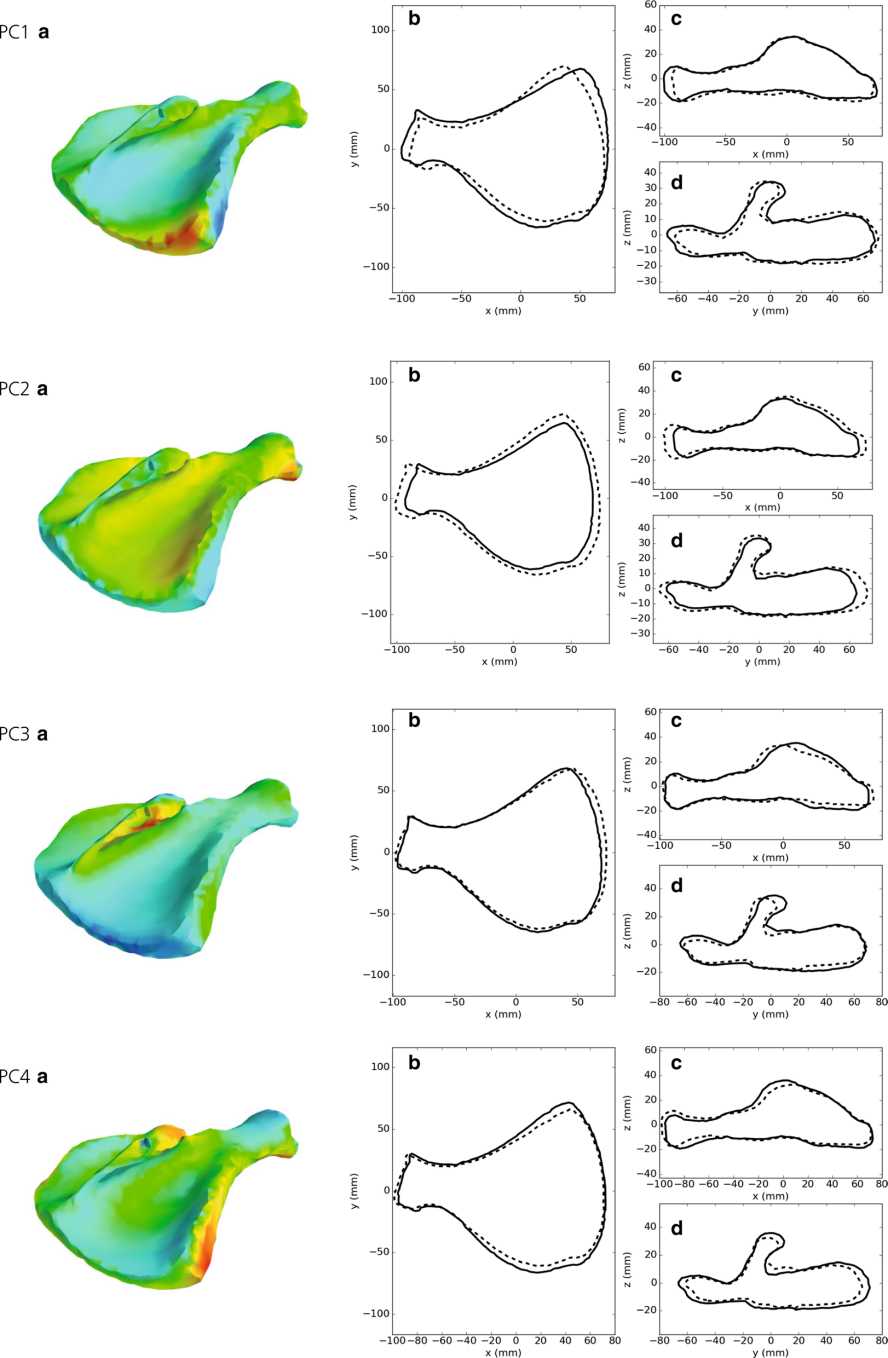
The first four principal components (PC) of the pig scapula. The colours of the 3D images indicate in what direction each PC altered the scapula shape. Red colour indicates growth perpendicular to the surface and blue colour indicates shrinkage perpendicular to the surface. The other plots show the contour of the scapula (x–y, x–z and y–z views) when adding (solid line) or subtracting (dotted line) two standard deviations of score values for each PC

Comparing the data from the first 10 PC with the input of PCA showed that using 10 PC (Fig. 3b) resulted in an average error of 1.4 mm for node positions. The effects of the first four PC are illustrated in Fig. 4. The first PC was associated with mass transfer between *infraspinous* and *supraspinous fossa* (see Fig. 1) and the curvature of the scapular spine in the y–z plane. The second PC was mainly dominated by size of the scapula. This can also be observed in the regression coefficients ($\beta$ from Eq. 2) in Table 1, where the regression coefficient for live weight was much larger for this component than for the others. The third PC was mainly associated with shape of the spina scapula and also affected curvature of the entire bone in the x–z plane. The fourth PC mainly scaled the length to width and the length to thickness ratios.

### Genetic parameters

To have a manageable number of traits in the genetic analysis, we focused on the first 10 PC and calculated genetic parameters using the DMU software [34]. Estimates of heritabilities of the PC were high (0.40–0.79) (Table 1), while estimates of the genetic correlation between PC ranged from − 0.2 to 0.25, with a standard error of ~ 0.1. Summing the product of the explained variance and heritability estimate for the 10 PC in Table 1 provides a rough estimate of the heritability of the observed scapula shape, and was equal to 0.47. However, this is a lower estimate, since variation in the remaining PC could also have a genetic component.

Each of the eigenvectors of the ${\mathbf{GP}}^{ - 1}$ matrix were associated mainly with one PC (Table 2). Ranking the eigenvectors by the size of their associated eigenvalue, the first eigenvector was dominated by PC8, which was the PC with the highest heritability (Table 1), while the subsequent eigenvectors were mainly associated with PC with gradually decreasing heritabilities. The main exceptions from this pattern were the third and fourth largest eigenvalues which are both, to some extent, associated with both PC3 and PC9.

**Table 2:** The ten largest eigenvectors (${\mathbf{v}}_{\varvec{n}}$) of the ${\mathbf{GP}}^{ - 1}$ matrix as linear combinations of principal components and the associated eigenvalues, $\lambda$

	${\mathbf{v}}_{1}$	${\mathbf{v}}_{2}$	${\mathbf{v}}_{3}$	${\mathbf{v}}_{4}$	${\mathbf{v}}_{5}$	${\mathbf{v}}_{6}$	${\mathbf{v}}_{7}$	${\mathbf{v}}_{8}$	${\mathbf{v}}_{9}$	${\mathbf{v}}_{10}$
${{\lambda }}$	0.80	0.79	0.69	0.68	0.67	0.63	0.56	0.53	0.51	0.40
PC1^a^	0.00	0.00	0.00	0.00	0.00	0.01	− 0.03	1.00	0.01	0.01
PC2	0.02	− 0.01	0.00	0.00	− 0.01	0.01	− 1.00	− 0.03	0.01	0.00
PC3	0.00	− 0.02	− 0.98	0.17	0.01	0.01	0.00	0.00	0.01	0.00
PC4	0.00	− 0.02	− 0.01	− 0.01	− 1.00	− 0.05	0.01	0.00	0.00	0.00
PC5	0.03	− 0.01	0.01	0.00	0.00	0.00	0.01	− 0.01	1.00	0.00
PC6	0.01	0.00	− 0.01	0.00	0.05	− 1.00	− 0.01	0.01	0.00	0.00
PC7	0.02	1.00	− 0.02	0.00	− 0.02	0.00	− 0.01	0.00	0.01	0.00
PC8	− 1.00	0.02	− 0.01	− 0.05	0.00	− 0.01	− 0.02	0.00	0.03	0.00
PC9	0.05	0.00	− 0.17	− 0.98	0.01	0.00	0.00	0.00	0.00	0.00
PC10	0.00	0.00	0.00	0.00	0.00	0.00	0.00	− 0.01	0.00	1.00

*PC* principal components of the 3D scapula shape

### Validation

**Fig. 5:**
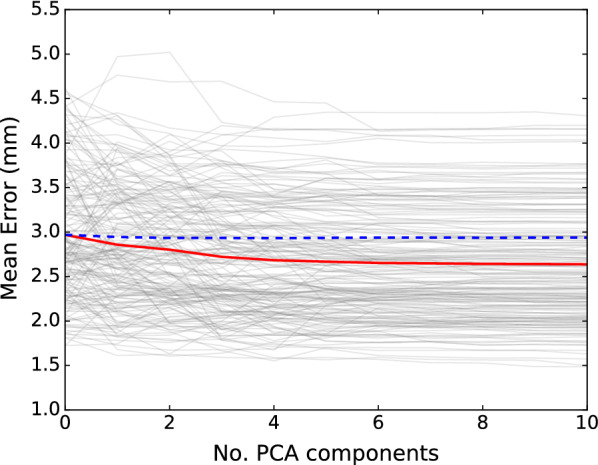
Mean error for prediction of pig scapula shape, when including genomic predictions based on the first 10 principal components. Grey lines represent the error for each validation animal. The red line represents the error mean across animals and the blue dotted line represents the mean error when only estimates of fixed effects were used for shape prediction

Of the 200 validation animals, 15 were removed because reliable estimates of fixed effects were not available. For the remaining 185 animals, estimates of fixed effects were added to the GEBV and then multiplied with the corresponding PC loadings (according to Eq. 4). Then, the predicted shape, $\hat{X}$ was compared with the true shape, $X$, based on the average Euclidean norm of all 1234 corresponding 3D-points (see Fig. 5). Adding predictions of more principal components reduced the mean error (across animals) from about 2.97 mm (when population average scapula was used as prediction) to 2.64 mm when 10 PC-predictions were used. For comparison, omitting the GEBV (in Eq. 2) and using only the fixed effect estimates (indicated by blue dotted line in Fig. 5) reduced the mean error from 2.97 to 2.94 mm, only.

**Fig. 6:**
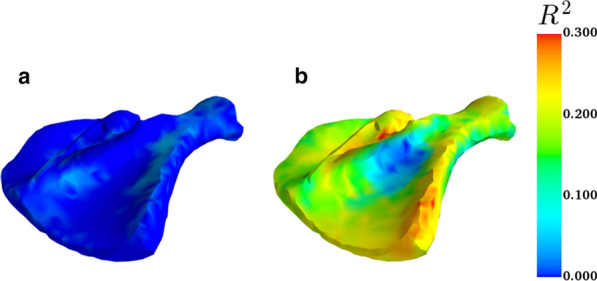
Spatial prediction reliability for the 3D model of the pig scapula based on fixed effects estimates only (**a**), and based on estimates of fixed effects plus genomic predictions, using the first 10 principal components (**b**)

The spatial prediction reliability, $R^{2}$, was calculated for each of the surface node points. The mean reliability for the full prediction model that included both GEBV and estimates of fixed effects was 0.18, with a standard deviation of 0.05 (Fig. 6b). By mapping reliabilities to the scapula, we see that the prediction reliability was slightly higher in convex than in concave regions. Specifically, some reliabilities were lower on the outside of the shoulder blade, close to the spine, both on the *infraspinous* and *supraspinous fossa* (see Fig. 6b). In comparison, using estimates of fixed effects only for shape prediction (Fig. 6a) resulted in spatial prediction reliabilities close to zero over the whole geometry.

## Discussion

In this paper, we present a computational pipeline for shape modelling of 3D surfaces of organs and a predictive model for 3D shape based on genomic data. In general, estimates of heritability (Table 1) were high (mean 0.62) compared to those of most traits related to skeletal features reported for humans [40–42], mice [43, 44], and livestock [45–47]. However, it should be mentioned that most of the literature on livestock focuses on bone size or on phenotypes related to bone length (e.g. height or stature), and not on bone shape, so the quantity of relevant data for comparison is limited. The high heritabilities of overall measures in the current study show that the shape of the scapula is highly heritable and that the computational pipeline that processes raw CT images to principal components is associated with limited errors.

In spite of the relatively small number of individuals used for training (1888), we have shown that genomic predictions of the 3D shape of the scapula was achievable. Adding additional principal components to predictions improved the average fit, resulting in an average error or predictions across points and animals of 2.64 mm based on the first 10 PC, which represents the sum of errors from genomic prediction and from simplification of the data when using 10 instead of 3702 variables. In addition, there are also some inherent imprecision in the CT data (voxel size of about 1 mm^3^), which limits the achievable predictive precision. Genomic predictions for shape had a mean reliability of 0.18, while predictions based on estimates of fixed effects only resulted in a prediction reliability close to zero. The reasons why estimates of genetics resulted in a better predictor of shape than estimates of environmental effects are: (1) the high heritability of the traits, and (2) the fact that the individuals were raised in very similar conditions and were scanned at a similar age/weight. For example, if the variation in body weight of scanned animals had been large, the predictive ability based on only estimates of fixed effects would probably have been higher.

The average spatial prediction reliability obtained here was of a similar order of magnitude as reported for facial shape predictions in humans [10]. In our data, reliability was quite spatially uniform, while for predictions of facial shape [10], reliability was relatively heterogeneous. In our data, the main area with lower spatial prediction reliabilities was on the outside of the shoulder blade, close to the spine, both on the *infraspinous* and *supraspinous fossa* side (blue regions in Fig. 6b). This is an area that has a slightly concave curvature in the y–z plane. In addition, the bone is quite thin in this region and the lowered prediction reliability might be because the CPD algorithm [29] led to conflicting correspondences between the subscapularis fossa’s surface and the *infraspinatus fossa*’s surface in some instances (see blue and green points in and corresponding curves in Fig. 2). This problem was also reported in an analysis of human scapula data [48], and in that study conflicting correspondences were handled first by dividing the surface into zones and then looking for correspondences between points belonging to the same zone, rather than fitting the whole point cloud in one step.

Previously, statistical shape models of scapula have been reported for humans [48–50], primates [51], and Felidae [52], but these were based on a much smaller number of individuals (between 15 and 57) than that used in our study. In addition, the scapula analysed here were probably more uniform in both size and shape than in these previous studies because the individuals were more homogeneous in terms of age and genetics and were raised under uniform environmental conditions. In some of these previous studies, the first principal component explained a large amount of the total variance of the data, i.e. 60% [50], 72% [49], and 99% [52], and represented mainly the size-component in the data. Even after removing the size-component from the total variation, fewer principal components were needed in these studies to explain a certain ratio of the total variance than the number required in our study.

Examination of genetically orthogonal linear combinations of the PC traits through eigen decomposition of the ${\mathbf{GP}}^{ - 1}$ matrix, showed almost a one-to-one relationship between PC traits and eigenvectors, which was as expected, since estimates of genetic correlations between the PC traits were relatively low (between − 0.2 and 0.25 with a standard error of ~ 0.1). The main exceptions to this one-to-one relationship were the third and fourth largest eigenvalues, which were both associated with the third and ninth principal component, which both had a heritability estimate equal to 0.68.

### Implications of the chosen methods

Our aim was to build a computational pipeline that could connect 3D data of an individual to its underlying genetics. Hence, the results rely on a variety of methods and assumptions, and some justification and implications of the chosen methods are presented here.

#### Creating the scapula atlas

In the current study, for simplicity, a random individual was selected to create the atlas for point correspondence. Other alternatives, such as using an average atlas or multi-atlas, have been shown to give a more accurate atlas [53, 54] and might have improved the point correspondence, especially in the concave regions and where the surfaces on different sides of the objects are close to each other.

#### Surface extraction method

In the current study, we used Delaunay triangularization and removed all tetrahedrons with any edge longer than a specified threshold. The rationale for this was to establish a more general method than, e.g., the marching cubes algorithm [27], which can also extract surfaces from less structured point clouds, such as those coming from 3D cameras. The level of the threshold chosen could, however, affect results. A high threshold could reduce the information about the concave areas, while a low threshold could result in a discontinuous surface. By using a threshold of 5 mm for a point cloud with a regular grid with a resolution equal to 0.94 × 0.94 × 1.25 mm, some smoothing occured in the most concave regions, as for example where the tendons attach to the scapular spine.

#### Point registration method

The CPD-algorithm was used to find corresponding points across individuals. Initial tests were conducted with default input parameters and the affine deformation was found to perform best. Optimization of parameters, which could potentially improve the achievements of the method, was not performed. In addition, as described in Methods, we used the most probable point correspondence (from matrix ${\mathbf{P}}$) for all points in X to find the corresponding point in Y. An alternative that could have improved the method is to use ${\mathbf{P}}$ to calculate weighted coordinate points from Y. A weakness of the original CPD-algorithm is that only the Euclidean distance is considered as a measure of similarity and not the neighbourhood structure of points [31], as exemplified by locating corresponding points on the wrong side of the object (Fig. 2). Much effort has been put into improving the CPD algorithm, such that it includes more information about the local structure between neighbouring points, see e.g. [31, 55]. Testing these developments as well as other relevant point registration methods [56, 57] is beyond the scope of this paper, but will be followed up in future research.

#### Scaling method

In the current study, the size of the object was embedded in the definition of the phenotypes. Before doing the PCA, the surface point cloud was rescaled to its original size, measured in mm. However, the PC-phenotypes were corrected for the live weight of the animal at scanning to account for different sizes of the animals and in order to keep the phenotypes as independent as possible from animal management decisions (e.g. day of scanning). An alternative for handling the effect of size would be to consider size separately from the PCA [58] and treat it as a separate scalar phenotype in the statistical model for estimating genetic parameters. Although this approach would have changed the principal components, the main conclusions would probably remain much the same, because the size component in the current dataset was relatively limited.

#### Training population and cross-validation

In the current study, a set of 200 animals was masked from the dataset before calculation of the principal components, estimation of genetic parameters, and genomic prediction. This was done because our aim was to demonstrate the concept of predicting the shape of an organ, rather than to obtain realistic prediction accuracies for animal breeding, which requires relevant relatedness between training and validation populations [59]. An alternative that would have increased the precision of the estimate of prediction reliability is to use K-fold cross-validation [60]. However, this would have changed the definition of the phenotypes and the estimates of genetic parameters between validation sets, which would have made the results more difficult to interpret.

### Further use of 3D animal models

Previously [4], we focused on the shape of the scapula spine and on some overall characteristics such as length, width, and thickness of the scapula. As hypothesized, we found that the shape of the scapula was genetically correlated with the severity of shoulder lesions and with body condition score of sows at weaning (of the litter). In the work reported here, we extended the shape modelling to capture the entire 3D geometry of the scapula, which allowed us to use all morphological variation from the CT-scans. This is important to better understand the relationships between morphology, health, and genetics. Furthermore, for animal breeding purposes, detailed 3D models could be used to develop relevant indicators for traits that are not possible to measure on selection candidates, such as longevity and robustness [61–63]. By including relevant precision phenotypes in multivariate genomic predictions, the accuracy of estimated breeding values for economically important traits will increase [5]. As such, the development of a statistical shape model for breeding can be important for animal welfare and efficient food production. In addition, the large number of CT scanned individuals in modern breeding programs, constitutes a valuable data source for verification of novel segmentation and detection algorithms. Furthermore, breeding animals are not covered by the General Data Protection Regulations, and high throughput genotyping and phenotyping from pig breeding could be used to improve reference datasets for genetic research across species, and to improve and automate diagnostic tools.

## Conclusions

In this work, we constructed a statistical shape model of the pig scapula, in which the first 10 principal components covered 80% of the total shape variance observed in a large population of CT-scanned pigs. In general, estimates of heritabilities of the first principal components were high and showed that the computational pipeline that processes raw CT data to principal component phenotypes was associated with little error. Furthermore, we showed that it is possible to predict the 3D shape of the scapula based on genomic data. Some limitations in the quality of the point correspondence were observed in concave areas where the surfaces of opposite sides of the scapula were close to each other. In those areas, the coherent point drift algorithm sometimes led to conflicting correspondences between the different sides of the bone, and the reliability of genomic predictions was lower than for the rest of the scapula. The statistical shape model can be used to develop precise indicator traits for phenotypes that are not possible to measure on selection candidates, such as longevity and robustness. As such, the development of detailed 3D animal models can be an important tool for improving animal welfare and for efficient food production.

## Data Availability

The data that support the findings of this study are available from Norsvin SA, but restrictions apply to the availability of these data, which were used under license for the current study, and thus are not publicly available. However, data are available from the authors upon reasonable request and with permission of Norsvin SA.
